# Cell‐type specific contribution to sex differences in Alzheimer’s Disease revealed using single‐cell transcriptomes

**DOI:** 10.1002/alz.092518

**Published:** 2025-01-03

**Authors:** Yiyang Wu, Logan C. Dumitrescu, Philip L. De Jager, Vilas Menon, Julie A. Schneider, David A. Bennett, Timothy J. Hohman

**Affiliations:** ^1^ Vanderbilt Memory & Alzheimer’s Center, Vanderbilt University Medical Center, Nashville, TN USA; ^2^ Vanderbilt Genetics Institute, Vanderbilt University Medical Center, Nashville, TN USA; ^3^ Center for Translational & Computational Neuroimmunology, Columbia University Irving Medical Center, New York, NY USA; ^4^ Taub Institute for Research on Alzheimer’s Disease and the Aging Brain, Columbia University Irving Medical Center, New York, NY USA; ^5^ Center for Translational & Computational Neuroimmunology, Department of Neurology, Columbia University Irving Medical Center, New York, NY USA; ^6^ Rush Alzheimer’s Disease Center, Rush University Medical Center, Chicago, IL USA; ^7^ Rush University, Chicago, IL USA; ^8^ Vanderbilt Memory & Alzheimer’s Center and Vanderbilt Genetics Institute, Vanderbilt University Medical Center, Nashville, TN USA

## Abstract

**Background:**

Two‐thirds of Alzheimer’s Disease (AD) cases are women, and our team has identified molecular factors that relate to disease in a sex‐specific manner. Here, we leverage single‐cell transcriptomics from dorsolateral prefrontal cortex (N = 424) from the Religious Orders Study and Memory and Aging Project (ROS/MAP; AD Knowledge Portal syn2580853) to characterize sex‐specific contributors at cellular resolution.

**Method:**

Single‐nucleic RNAseq data was generated and processed as previously described. Sex‐stratified and sex interaction analyses were performed by fitting negative binomial mixed models (‘Nebula’ R package) in relation to AD diagnosis, AD pathology, and cognitive decline. We also conducted gene set enrichment (‘fgsea’ R package) and global and neural‐specific cell‐cell communication profiling (‘CellChat’ and ‘NeuronChat’ R package). Correction for multiple comparisons was completed with the false discovery rate procedure.

**Result:**

68% samples were females, and 37% were AD cases. We identified 85 sex‐specific effects that had FDR significant interaction effects and FDR significant stratified effects in one sex (31 for cognition, 28 for amyloid burden, and 27 for tangle burden). Most male‐specific effects were observed in endothelial cells, while female‐specific effects tended to be in excitatory neurons. Interestingly, among females higher *ADGRG7* across all cell types related to more plaques and tangles, while in contract, in males’ neuronal expression of *ADGRG7* was associated with lower tangle burden. Of the 85 sex‐specific effects, many have not been identified previously. Two signaling pathways had significant sex‐opposite gene enrichment effects including ‘TNFA_SIGNALING_VIA_NFKB’ for cross‐sectional cognition in microglia, and ‘OXIDATIVE_PHOSPHORYLATION’ for tangle burden in astrocytes and endothelial cells, with males enriched in upregulated genes and females enriched for downregulated genes. Compared to males with AD, female counterparts showed a significant reduction in global intercellular communication strength, particularly for incoming signals from inhibitory neurons and outgoing signals from excitatory neurons. Nevertheless, females with AD had more cell‐cell communication signals that were lacking in males with AD, such as THY1, GRN, VEGF, and COMPLEMENT signaling.

**Conclusion:**

We identified dozens of novel sex‐specific molecular associations and interesting cell‐cell communication pattern differences between sexes. Future mechanistic studies should connect these genes with their downstream effectors in a cell‐type specific manner.

